# Trends in life expectancy: did the gap between the healthy and the ill widen or close?

**DOI:** 10.1186/s12916-020-01514-z

**Published:** 2020-03-20

**Authors:** Anna C. Meyer, Sven Drefahl, Anders Ahlbom, Mats Lambe, Karin Modig

**Affiliations:** 1 Unit of Epidemiology, Institute of Environmental Medicine, Karolinska Institutet, PO Box 210, SE-171 77 Stockholm, Sweden; 2 Demography Unit, Stockholm University, SE-10691 Stockholm, Sweden; 3 Department of Medical Epidemiology and Biostatistics (MEB), Karolinska Institutet, SE-17177 Stockholm, Sweden; 4 Regional Cancer Centre, University Hospital, SE-751 85 Uppsala, Sweden

**Keywords:** Life expectancy, Population aging, Population health, Epidemiology, Education, Cancer, Cardiovascular diseases

## Abstract

**Background:**

During the past decades, life expectancy has continued to increase in most high-income countries. Previous research suggests that improvements in life expectancy have primarily been driven by advances at the upper end of the health distribution, while parts of the population have lagged behind. Using data from the entire Swedish population, this study aims to examine the life expectancy development among subgroups of individuals with a history of common diseases relative to that of the general population.

**Methods:**

The remaining life expectancy at age 65 was estimated for each year in 1998–2017 among individuals with a history of disease, and for the total Swedish population. We defined population subgroups as individuals with a history of myocardial infarction, ischemic or hemorrhagic stroke, hip fracture, or colon, breast, or lung cancer. We further distinguished between different educational levels and Charlson comorbidity index scores.

**Results:**

Life expectancy gains have been larger for men and women with a history of myocardial infarction, ischemic or hemorrhagic stroke, and colon or breast cancer than for the general population. The life expectancy gap between individuals with a history of hip fracture or lung cancer and the general population has, however, been growing. Education and comorbidity have affected mortality levels, but have not altered the rate of increase in life expectancy among individuals with disease history. The female advantage in life expectancy was less pronounced among individuals with disease history than among the general population.

**Conclusions:**

Life expectancy has increased faster in many subpopulations with a history of disease than in the general population, while still remaining at lower levels. Improvements in life expectancy have been observed regardless of comorbidity or educational level. These findings suggest that the rise in overall life expectancy reflects more than just improved survival among the healthy or the delayed onset of disease.

## Background

Human life expectancy has increased by more than 2 years per decade during the past century in Sweden as well as many other high-income countries [1, 2]. The question of whether the increase in life expectancy has occurred at a similar pace across population groups has been investigated in a number of studies. In such studies, the focus has often been on the specific question of whether parts of the population remain disadvantaged and are left behind as life expectancy increases [3–10]. Previous research has suggested that improvements in health in recent years have been driven by advances at the upper end of the health distribution, while other parts of the population have lagged behind [11–13]. Although these studies did not estimate life expectancy, their results could indicate that the increase in life expectancy has primarily been driven by the healthy part of the population and that those affected by disease experienced slower increases in life expectancy than the general population.

At the same time, the advances in survival from cardiovascular diseases and several cancer types have been substantial throughout high-income countries [14–20]. However, improved rates of survival from disease do not necessarily correspond to a longer life expectancy among survivors beyond the acute phase. Most existing epidemiological studies estimate survival rates following a *real* cohort of patients, and only a few utilize period life table techniques. One major limitation of observing real cohorts is the inability to describe recent developments. Given that survival chances continuously improve for many diseases, survival estimates based on older cohorts provide overly pessimistic scenarios that potentially overshadow more favorable survival trends in recent years. In this study, we calculate the remaining life expectancy among population groups with a history of common and severe diseases and examine how it has developed over time. We then compare these trends to the development of life expectancy in the total population.

We are not aware of any existing study that has examined trends in the remaining life expectancy for populations with a history of hip fracture, myocardial infarction, or stroke—even though the latter two conditions are the most common causes of death both in Sweden and globally [21]. A few studies that have explored trends in life expectancy among population subgroups with, for instance, diabetes or some cancer types have reported converging trends, i.e., a narrowing gap between life expectancy in the general population and among subgroups with a history of disease [22, 23]. A persistent gap was, however, found for population subgroups with lung cancer [23].

By using register data covering the total Swedish population, our aim is to examine whether the remaining life expectancy of men and women who have experienced common and severe diseases has improved at a pace similar to that of the general population. We have included myocardial infarction, stroke, hip fracture, and certain types of cancer since these conditions are among the most common diseases in old age, can be reliably studied in population registers, and cover a broad disease panorama. We estimate the gap in the remaining life expectancy between population groups with a history of these diseases and the general Swedish population among men and women separately and examine how this gap has changed between 1998 and 2017. We additionally stratify the analyses by educational and comorbidity levels in order to examine whether life expectancy trends in populations with disease history are sensitive to these two factors, since both education [4, 5, 24] and comorbidity [17, 25] are known to substantially impact mortality.

## Methods

### Data sources and study population

Through record linkage between several population registers using the personal identity number assigned to all residents of Sweden, the total Swedish population, as well as individuals with a disease diagnosis, could be identified. The linkage was conducted by Statistics Sweden, and the researchers were given an anonymized dataset. All data sources are described in Supplementary Figure 1. The date and cause of each death were retrieved from the Cause of Death Register, and disease diagnoses were obtained from the National Patient Register (NPR) (myocardial infarction, ischemic stroke, hemorrhagic stroke, and hip fracture) or the Cancer Register (colon, female breast, and lung cancer). The NPR contains data on all hospitalizations in Sweden. The register also provides particularly good coverage for these diseases, because they almost always require hospitalization, have a clear onset, and are usually accurately diagnosed [26, 27]. As reporting to the Swedish Cancer register is mandated by law, the register has an estimated completeness exceeding 95% [28]. The categories used to indicate the highest educational level achieved are basic education (up to 8–9 years of compulsory education, depending on the birth cohort) and more than basic education. The Charlson comorbidity index (CCI) scores [29, 30] were calculated based on diagnoses made during previous hospital admissions. The primary and secondary diagnoses at first disease occurrence, and in the 1-year period leading up to the first disease occurrence, were taken into account. All data were available up to 31 December 2017, except for the Cancer Register data, which were available up to the end of calendar year 2016.

The following ICD codes were used to identify primary diagnoses for the diseases of interest: ischemic stroke 433-34 (ICD-9), I63 (ICD-10); hemorrhagic stroke 431-32 (ICD-9), I61-62 (ICD-10); myocardial infarction 410 (ICD-9), I21-22 (ICD-10); hip fracture 820 (ICD-9) and S72, excluding codes S72.3, S72.4, S72.9 (ICD-10); colon cancer 153 (ICD-7), C18 (ICD-10); lung cancer 162-63 (ICD-7), C33-34 (ICD-10); and female breast cancer 170 (ICD-7), C50 (ICD-10).

### Life expectancy calculation

The remaining life expectancy at age 65 was estimated for all calendar years between 1998 and 2017 in the general population and in each subpopulation of individuals with a history of disease. Individuals entered the study population on their 65th birthday and entered a disease subpopulation when they received a disease diagnosis. Person-years and deaths observed in a single year were used to estimate the remaining period life expectancy as the area under the Kaplan-Meier product limit estimator [31]. This procedure is equivalent to the period life table technique used by the WHO [32], but it can take advantage of the continuous information available in Swedish registers. To compare life expectancy between subpopulations and the general population, absolute differences in years were calculated.

For each subpopulation with a history of disease, the remaining life expectancy was further estimated separately for individuals with basic and higher education and for individuals with CCI scores of 0, 1, and > 1, except for cancer and hemorrhagic stroke cases, for which two categories were used due to the small numbers of individuals diagnosed with these diseases. To ensure that the number of cases was sufficient, the study period was additionally aggregated into five calendar-year groups. All models were stratified by gender, and the analyses were conducted in Stata version 14.2 and R version 3.3.1.

### Sensitivity analyses

The estimated age-specific mortality rates in subpopulations with a history of disease could be affected by an unequal distribution of age at disease onset over time. Holding age at disease onset constant eliminates this potential compositional influence. Thus, in sensitivity analyses, we compared the remaining life expectancy among individuals with a fixed age of disease onset (age 75 for cancers and age 80 for the other diseases) and among those who remained disease-free at this age. In these sensitivity analyses, the populations with disease history comprised only individuals with disease onset at the exact age of 80 (age 75 for cancers).

Mortality shortly after disease onset is high for individuals experiencing a myocardial infarction, stroke, and hip fracture. Therefore, in order to examine the impact of mortality improvements in the acute phase, we estimated the remaining life expectancy excluding those who died within 28 days after disease onset. Since mortality for cancer does not show a similar pattern of increased mortality close to disease onset, we did not include cancer in these analyses. Finally, to explore if trends in life expectancy are sensitive to changes of the baseline age, we estimated the remaining life expectancy also at ages 60, 75, and 85 years.

## Results

A total of 3,877,437 individuals were included in our study, of whom 53% were women and 47% were men. Of these individuals, approximately 14% had a recorded event of myocardial infarction, 11% had an ischemic stroke, 10% had a hip fracture, and 2% had a hemorrhagic stroke. In addition, 8% of the women were diagnosed with breast cancer, while lung and colon cancer were diagnosed among 3% and 2% of the study population, respectively. The number of men and women in the general Swedish population and each subpopulation together with the mean age of disease onset are provided in Supplementary Table 1 for the years 1998, 2008, and 2017.

Data on education were available for 95% of the study population and indicated that 56% had attained more than basic education. Among the individuals diagnosed with any of the diseases, 36% had a CCI score of least 1 and 13% had a CCI score of at least 2.

### Trends in remaining life expectancy

**Fig. 1:**
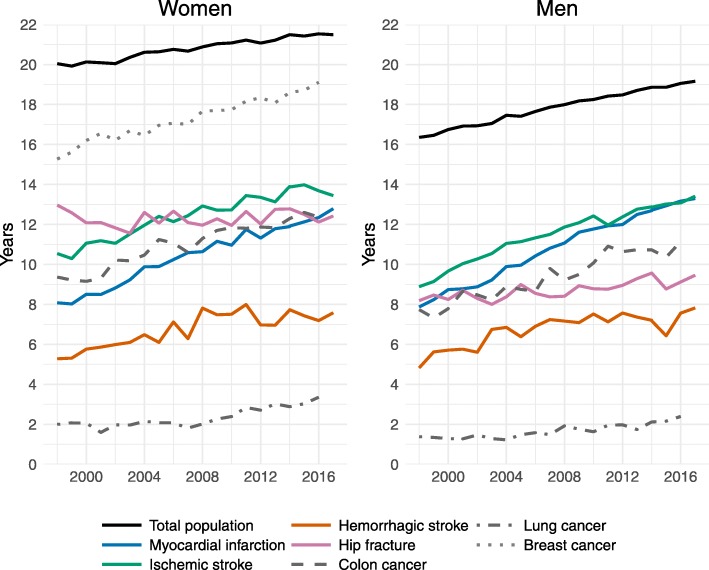
Trends in the remaining life expectancy at age 65 for subpopulations with a history of disease and the general Swedish population, 1998–2017

During the 20-year study period, the remaining life expectancy at age 65 rose by approximately 9 months per decade among women and 1.5 years per decade among men, reaching 21.5 years for women and 19.2 years for men in 2017 (Fig. 1, Table 1).

**Table 1:** Average pace of changes in the remaining life expectancy at age 65 among the general population and among individuals with a history of disease, 1998–2017

	Pace of RLE change (years/decade)	
	Women	Men
General population	0.76	1.48
Myocardial infarction	2.48	2.86
Ischemic stroke	1.53	2.38
Hemorrhagic stroke	1.21	1.58
Hip fracture	− 0.29	0.67
Colon cancer^a^	1.67	1.89
Lung cancer^a^	0.75	0.54
Breast cancer^a^	2.13	–

*RLE* remaining life expectancy at age 65

^a^Calculated for the 1998–2016 period

The remaining life expectancy at age 65 was lower in subpopulations with a history of disease than in the general population. At the end of the study period, life expectancy was lowest among those with a history of lung cancer (3.4 years for women, 2.4 years for men) and was highest among women who had a history of breast cancer (19.1 years) (Fig. 1). In all subpopulations, except for women who had experienced a hip fracture, life expectancy increased over time. Life expectancy rose faster among women and men with a history of myocardial infarction, ischemic stroke, hemorrhagic stroke, colon cancer, and breast cancer than it did among the general population (Table 1). In these subpopulations, increases in the remaining life expectancy per decade ranged from 1.2 years (women with hemorrhagic stroke) to 2.9 years (men with myocardial infarction).

The gaps in the remaining life expectancy between these subpopulations and the general population declined most notably for women and men who had experienced myocardial infarction or ischemic stroke and for women with a record of colon or breast cancer (Table 2). Reductions were smaller among women and men with a history of hemorrhagic stroke and among men who had colon cancer. By contrast, the gaps widened for subpopulations with a history of lung cancer and hip fracture.

**Table 2:** Differences in the remaining life expectancy (in years) between populations with a history of disease and the general population, and the changes in these differences, 1998–2017

	Women			Men		
	1998	2017	Change	1998	2017	Change
Myocardial infarction	11.96	8.70	− 3.26	8.48	5.86	− 2.62
Ischemic stroke	9.50	8.05	− 1.45	7.47	5.75	− 1.72
Hemorrhagic stroke	14.76	13.91	− 0.85	11.53	11.34	− 0.19
Hip fracture	7.08	9.07	+ 1.99	8.17	9.70	+ 1.54
Colon cancer	10.67	9.17^a^	− 1.51	8.62	7.91^a^	− 0.71
Lung cancer	18.04	18.18^a^	+ 0.13	14.98	16.66^a^	+ 1.68
Breast cancer	4.77	2.43^a^	− 2.35	–	–	–

^a^Data for year 2016

### Gender differences

Among the general Swedish population, women had 4 more years of remaining life expectancy than men in 1998, though this gap had declined to 2.5 years in 2017 (Fig. 1). Among the subpopulations who had a history of disease, the gender differences in remaining life expectancy were smaller. Thus, the gap in the remaining life expectancy between the general population and the subpopulations was larger for women than for men (Table 2). One exception was the hip fracture population, among whom the gap was wider for men than for women. Among the subpopulations who experienced cardiovascular diseases, women did not have a higher life expectancy than men. Indeed, the findings indicated that, compared to women, men with a history of myocardial infarction or hemorrhagic stroke had a life expectancy in 2017 that was 6 months higher and 3 months higher, respectively (Fig. 1).

### Education and comorbidities

**Fig. 2:**
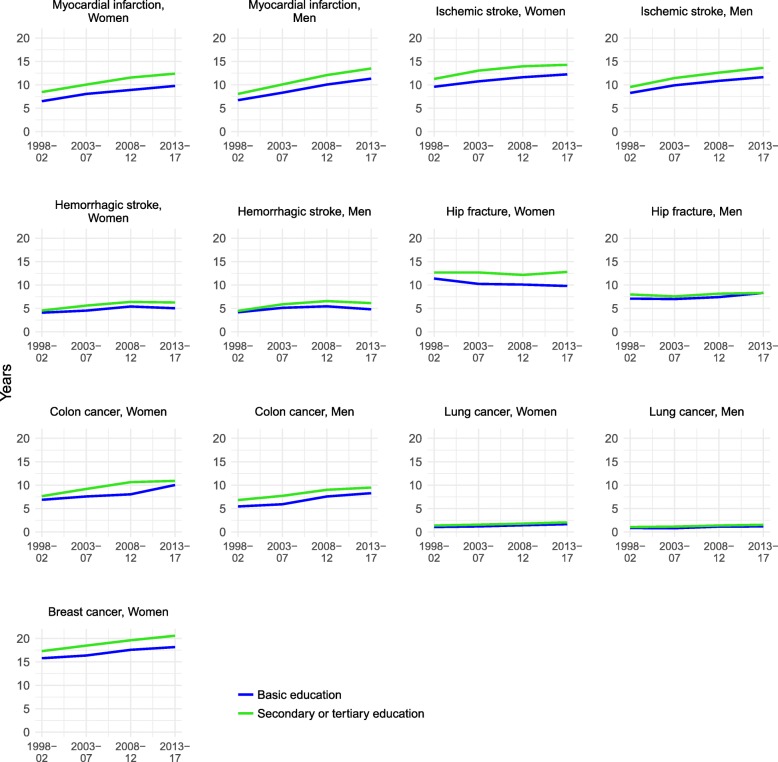
Trends in the remaining life expectancy at age 65 among subpopulations with a history of disease stratified by educational level and gender, 1998–2017

In all subgroups affected by disease, the remaining life expectancy was lower among men and women with basic education than it was among those with higher education (Fig. 2). Nevertheless, improvements occurred at a similar pace regardless of educational level.

**Fig. 3:**
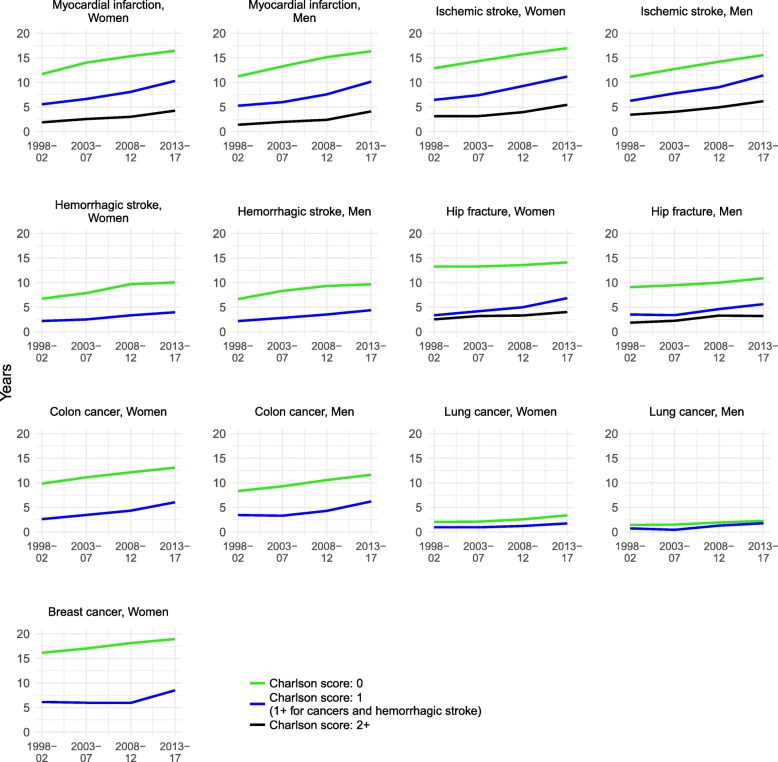
Trends in the remaining life expectancy at age 65 among subpopulations with a history of disease stratified by Charlson comorbidity index score and gender, 1998–2017

In all disease subpopulations, a high comorbidity burden was clearly associated with shorter life expectancy (Fig. 3). In 2013–2017, for instance, both women and men with a history of myocardial infarction and a CCI score of 0 reached a remaining life expectancy of more than 16 years, while those with a comorbidity score of > 1 had a life expectancy of about 4 years. Still, the remaining life expectancy rose across all subgroups distinguished by CCI score, and for all diseases. In the main analyses, we detected no increases in life expectancy among women with a history of hip fracture, and only small improvements among men. However, stratification by comorbidity score revealed upward trends in all strata. There was no evidence of a slower or a faster pace of improvements among healthier individuals, i.e., among those with no record of comorbidities.

### Sensitivity analyses

First, we estimated the remaining life expectancy at a constant age of disease onset. This yielded results similar to those of the main analyses (Supplementary Figure 2). Next, for myocardial infarction, stroke, and hip fracture, all individuals who died within 28 days of their disease event were excluded. This, too, did not notably change the results (Supplementary Figure 2). The pace of life expectancy improvements was faster among subgroups affected by myocardial infarction, ischemic stroke, colon cancer, or breast cancer than it was among the populations free of the respective disease, but it was slower among the subpopulations with a hip fracture or lung cancer. The only diverging pattern was found for those who had a hemorrhagic stroke: while our main analyses indicated that the gap in the remaining life expectancy between these individuals and the general population was shrinking, the sensitivity analyses found that this gap was increasing slightly. Absolute gains in life expectancy were generally smaller in the sensitivity analyses, which was expected because of the higher baseline age. The gender differences were small, except for the hip fracture and the colon cancer populations, among whom women had a remaining life expectancy that was roughly 2 years higher than that of men. Finally, changing the baseline age of life expectancy calculation to 60, 75, and 85 years revealed trends in life expectancy similar to our main analyses (Supplementary Figure 3).

## Discussion

While medical progress has contributed to prolonged survival following the onset of several diseases, it has been pointed out that such advances may have enabled increasingly frail individuals to survive to higher ages [33, 34]. Despite improved survival, these individuals may eventually face a higher risk of death at older ages and consequently have a lower life expectancy compared to their healthy counterparts [33, 34]. This study does not support this concern. In fact, we found that life expectancy rose faster in several population subgroups with a history of disease than it did in the general population, albeit while remaining at lower levels. Taken together, our results indicate that rising life expectancy is, at least in part, a result of higher life expectancy in subpopulations with disease history. Moreover, the improvements in life expectancy can be attributed not only to primary prevention efforts—i.e., preventing diseases from occurring or delaying the onset of diseases to higher ages—but also to secondary and tertiary prevention efforts that enable more patients to survive in the long term.

Our results are consistent with previous findings of considerable improvements in survival rates among patients with cardiovascular diseases [15, 17, 35] and stable survival rates among hip fracture patients [36, 37]. The rapid increases in remaining life expectancy found among subpopulations with a history of colon or breast cancer, and the smaller improvements found among those with a history of lung cancer, also correspond to previously observed trends in cancer survival [16, 23]. However, none of these studies estimated remaining life expectancy.

The underlying explanations for the trends observed in this study are likely multifactorial. Even if this study aimed at describing rather than explaining such trends, our results suggest some potential explanations. The advances in life expectancy among subpopulations with a history of myocardial infarction, stroke, or hip fracture observed in this study partly reflect the increased survival of patients in the acute phases of diseases through, for instance, improved surgical methods or higher proportions of patients reaching the hospital in time. Nevertheless, excluding from our analyses individuals who died within 28 days of disease onset did not notably change the pace of increases. This suggests that improvements in the acute setting are not the only underlying causes of rising life expectancy.

The improvements in life expectancy over the study period were not uniform across the different subgroups in this study. While gains in life expectancy among men and women with a history of myocardial infarction, stroke, colon cancer, or breast cancer surpassed those among the general population, improvements among individuals with a history of hip fracture and lung cancer were slow to non-existent. The lack of increases in life expectancy in the hip fracture population is striking. A recent study has attributed the stagnation in the length of survival after hip fracture to a higher prevalence of comorbidities [37]. In line with this observation, we found some increases in the remaining life expectancy among individuals with a history of hip fracture when stratifying by comorbidity level, which indicates that comorbidity has confounded survival trends in the hip fracture population. Nevertheless, rising comorbidity levels have also been reported for individuals with myocardial infarction and stroke [18, 20, 38], among whom life expectancy increased markedly, even without taking comorbidity into account. The prevalence of multi-morbidity among the old is rising in most developed countries [39–41] as a result of improvements in survival after the onset of disease, and of increasing longevity in general. However, we found that life expectancy rose in most of our disease populations, and among individuals with both low and high levels of comorbidity. Moreover, life expectancy in populations with disease history increased at a similar pace across educational levels, which suggests that the improvements were not limited to the highly educated or to those who were comparatively healthy when they were first affected by disease.

Women live longer than men in almost all human populations [42]. For example, in the Swedish population in 2017, life expectancy at age 65 was more than 2 years higher among women than among men. However, the gender differences among subpopulations with a history of disease were smaller. For cardiovascular diseases in particular, there were no notable gender differences in remaining life expectancy. As a consequence, the gap in life expectancy between individuals with a disease background and the general population was larger for females than for males. In 2017, for instance, the gap in life expectancy at age 65 between individuals with a history of myocardial infarction and the general population was roughly 9 years among women but only 6 years among men. Men experienced faster improvements than women, both in the general population and in most subgroups with disease history. Thus, while the gap in life expectancy compared to the general population was larger among women, they also experienced slower improvements compared to men. Whether these disparities reflect differences in the characteristics of the affected women and women in the general population; heterogeneity in disease manifestations, such as differences in severity between men and women; or differential survival chances cannot be answered with our data. Still, the larger gender differences in the general population than among the subgroups with disease history appear—at least to some extent—reasonable given that women’s lower risk of cardiovascular diseases [43, 44] is one reason for the higher life expectancy of females in the general population. Results from previous studies have further shown that even if women have lower risks of incident myocardial infarction or stroke than men, the risks of recurrent events or mortality barely differ between affected women and men [43, 44]. This suggests that the higher life expectancy among women than among men may be more closely linked to disease risk than to prognosis once a disease has occurred.

Period life expectancy summarizes age-specific mortality rates observed in a fixed time period and reflects the hypothetical mean life span of individuals assuming that mortality rates remain constant in the future. As such, life expectancy is influenced by the population composition which may change over time [45]. Numerous factors, such as migration, or social and medical conditions today and in the past shape both the general population and populations with disease history. In populations with disease history, age at (and time since) disease onset is an important predictor of mortality [45]. Therefore, we performed sensitivity analyses holding age at disease onset constant which yielded similar findings.

This study is based on data from the entire Swedish population, including frail and disadvantaged population groups. The Swedish population registers have high levels of completeness and validity [26–28], and the diseases we examined are among the most common medical conditions that occur at older ages, not only in Sweden, but in many other countries with a similar age structure and disease panorama. During the period under study, no screening programs were introduced for the cancer types included in our analyses. Still, attendance rates in the existing screening program for breast cancer may have changed over time influencing the timing of breast cancer diagnosis. Furthermore, public awareness, diagnostic sensitivity, and precision have likely improved over time and thus could have led to the earlier detection of cancer and consequently to improved survival. Therefore, we cannot rule out that trends in life expectancy among cancer populations may be influenced by lead time bias, i.e., a trend towards earlier detection of diseases, and thus towards cancers being less advanced at diagnosis. This may be part of an explanation for the rising life expectancy of men and women with a history of cancer.

It should also be noted that the CCI cannot identify all prevalent comorbidities; its specificity is not perfect. It is, therefore, likely that parts of our study population had higher comorbidity levels than their CCI scores suggest. This may have led to some dilution of the differences between comorbidity levels and to an underestimation of life expectancy in subgroups without comorbidities.

In our data, we could not identify population subgroups with diabetes or dementia, even though these conditions constitute a tremendous burden in aging societies. Future studies should examine trends in life expectancy following the onset of these diseases. Moreover, future research should investigate the mechanisms behind the observed differences in disease-specific trends, such as the lack of improvements in life expectancy among men and women with a history of hip fracture.

## Conclusion

Remaining life expectancy has increased faster in subpopulations with a history of disease than in the general population, while still remaining at lower levels. This finding suggests that improvements in overall life expectancy are not limited to the healthy population. In addition, it indicates that improvements are attributable not only to primary prevention—that is, preventing diseases from occurring or postponing their onset to higher ages—but to higher life expectancy levels among those who have been affected by disease. The well-documented female advantage in life expectancy was found to be less pronounced in subpopulations with a disease history than in the general population.

## Supplementary information

**Additional file 1: Supplementary Figure 1.** Data sources linked through personal identification numbers and years available.

**Additional file 2: Supplementary Figure 2.** Trends in remaining life expectancy of disease-free individuals and individuals with disease onset at age 80 or 75 by disease and gender in 1998–2002, 2003–2007, 2008–2012, and 2013–2017.

**Additional file 3: Supplementary Figure 3.** Trends in remaining life expectancy at age 60, 75, and 85 for subpopulations with a history of disease and the general Swedish population, 1998–2017.

**Additional file 4: Table S1.** Number of men and women in the total population and in subpopulations with disease history as well as mean age of disease onset in 1998, 2008, and 2017.

## Data Availability

The data sets generated and analyzed for this study were provided by the Swedish National Board of Health and Welfare and Statistics Sweden. The data were used under license for the current study. Restrictions apply to the availability of these data, which are thus not publicly accessible. Data are, however, available from the authors upon reasonable request and with permission of the regional ethics board in Stockholm. Statistical code is available upon request from the corresponding author at anna.meyer@ki.se.
