# Supplementary figures and images for: Trends in life expectancy: did the gap between the healthy and the ill widen or close?
Source: BMC Med. 2020 Mar 20;18:41. doi: 10.1186/s12916-020-01514-z (PMC7082956; doi:10.1186/s12916-020-01514-z)

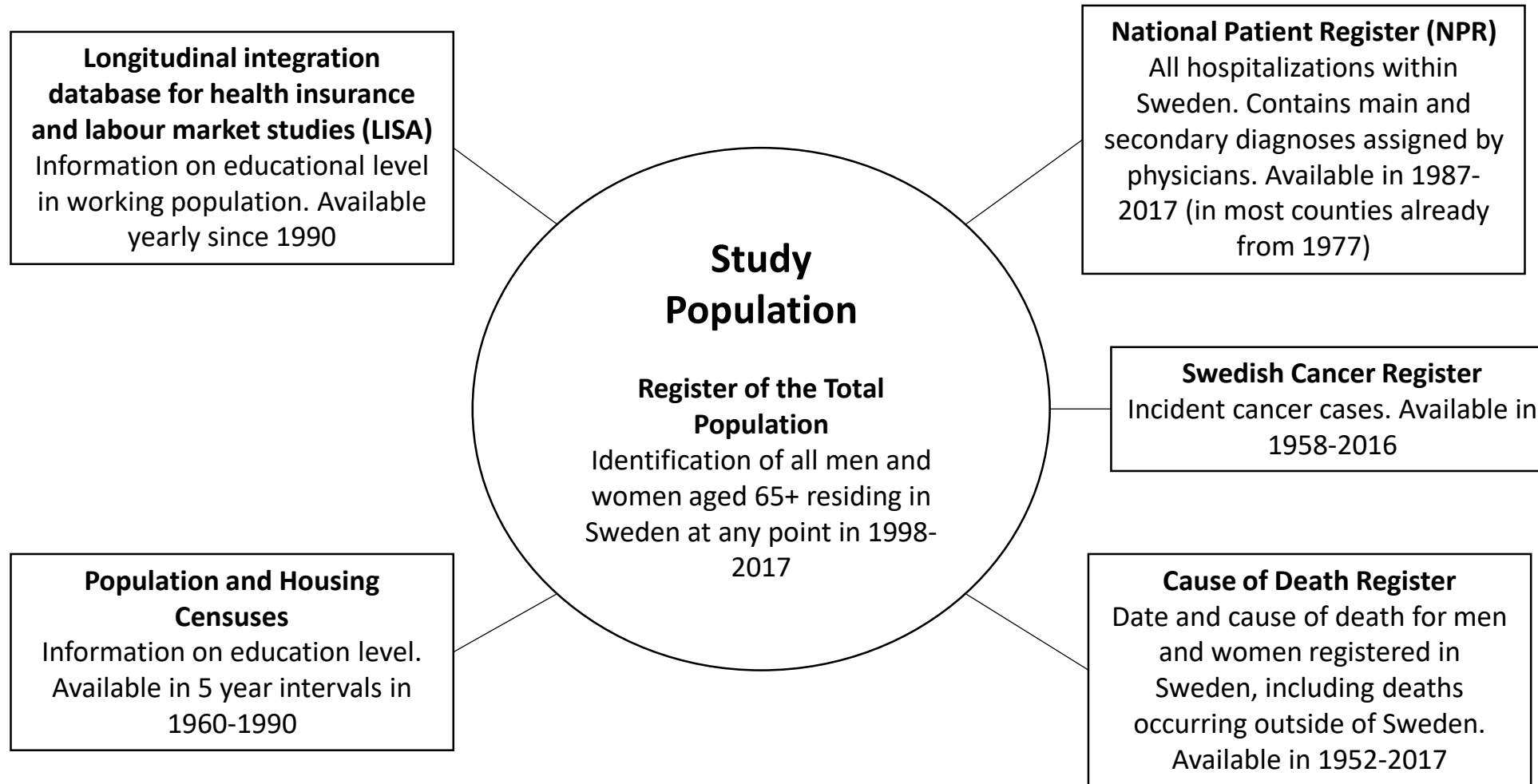

Supplement: Supplementary file 1 — Additional file 1: Supplementary Figure 1. Data sources linked through personal identification numbers and years available. [file 12916_2020_1514_MOESM1_ESM.pdf]

Years

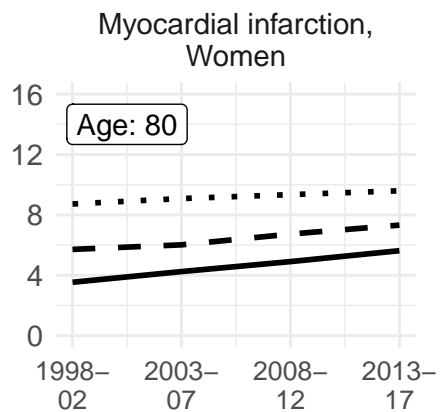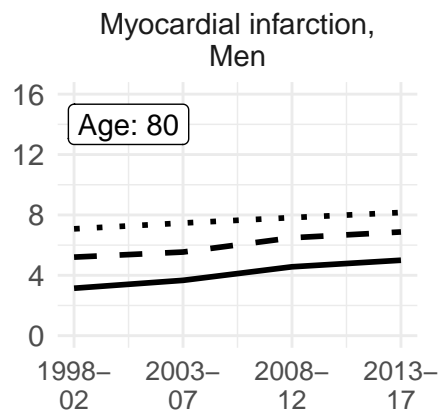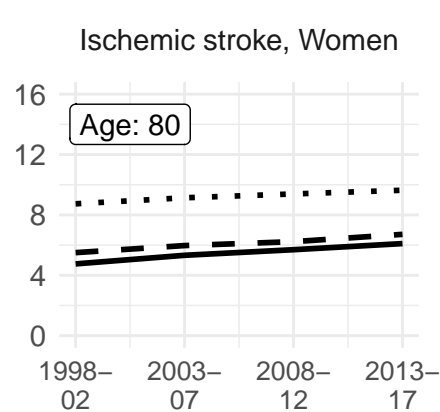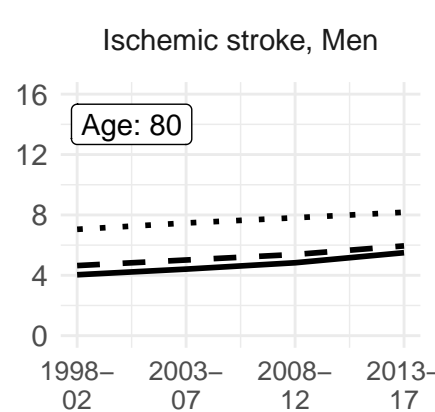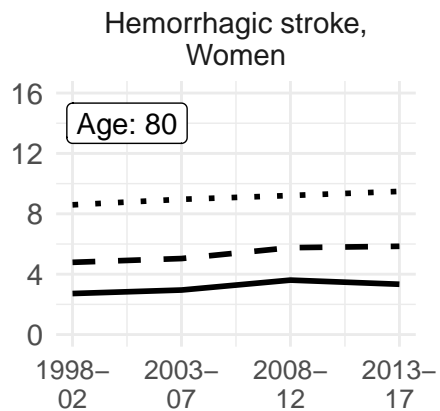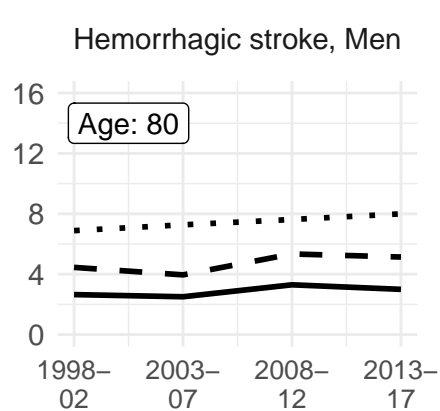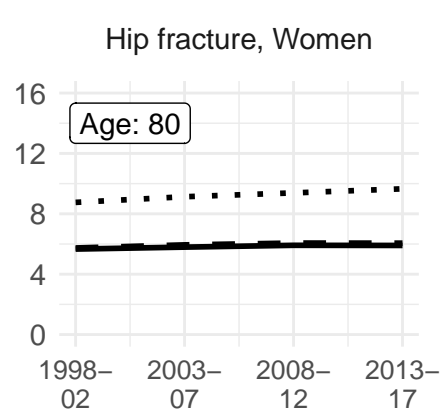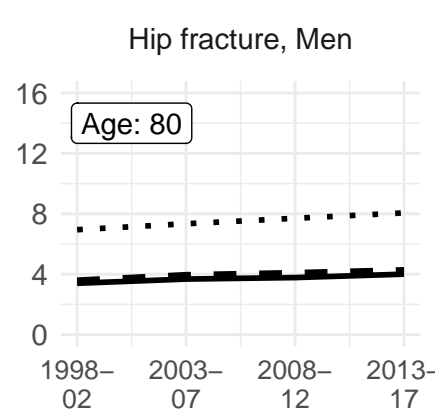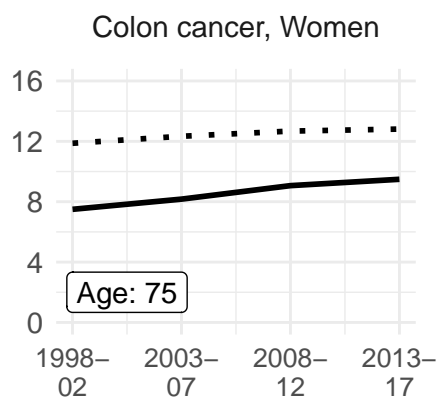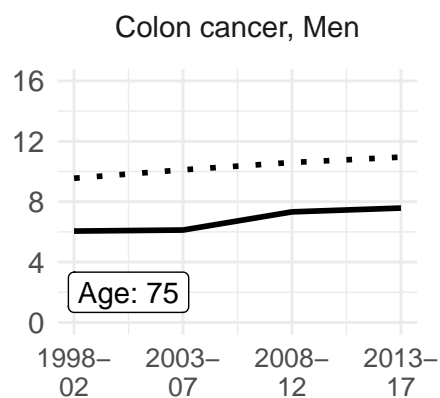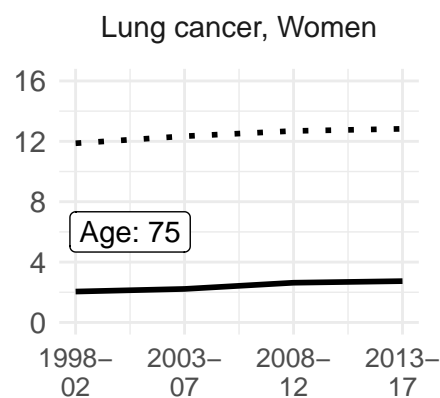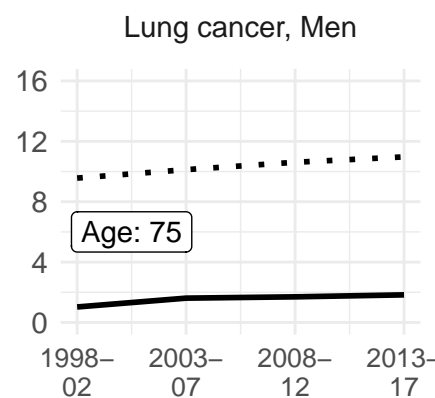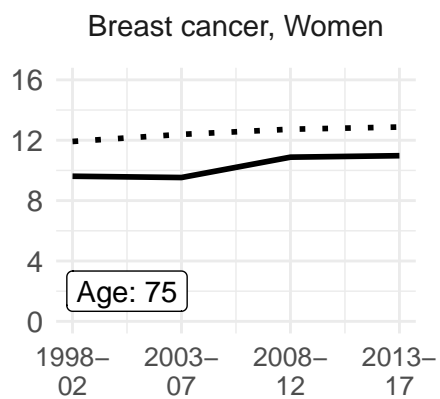

— All affected by disease  
- - - Affected by disease and surviving 28 days  
... Disease-free population

Supplement: Supplementary file 2 — Additional file 2: Supplementary Figure 2. Trends in remaining life expectancy of disease-free individuals and individuals with disease onset at age 80 or 75 by disease and gender in 1998–2002, 2003–2007, 2008–2012, and 2013–2017. [file 12916_2020_1514_MOESM2_ESM.pdf]
