# Supplementary material for: Trends in life expectancy: did the gap between the healthy and the ill widen or close?
Source: BMC Med. 2020 Mar 20;18:41. doi: 10.1186/s12916-020-01514-z (PMC7082956; doi:10.1186/s12916-020-01514-z)
Supplement: Supplementary file 3 — Additional file 3: Supplementary Figure 3. Trends in remaining life expectancy at age 60, 75, and 85 for subpopulations with a history of disease and the general Swedish population, 1998–2017. [file 12916_2020_1514_MOESM3_ESM.pdf]

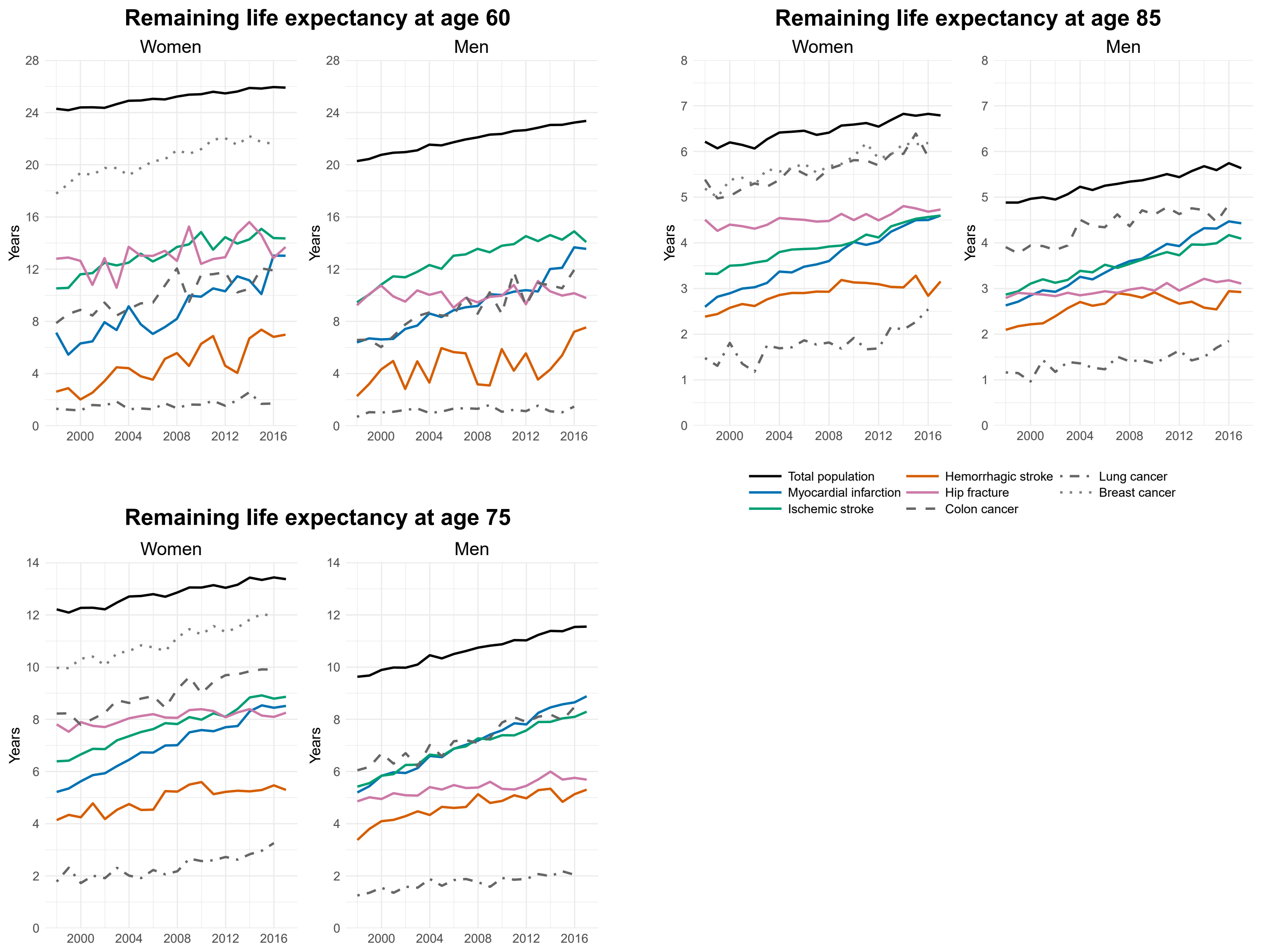

Remaining life expectancy at age 60
Women
Men
Years
0
4
8
12
16
20
24
28
2000
2004
2008
2012
2016
Remaining life expectancy at age 85
Women
Men
Years
0
1
2
3
4
5
6
7
8
2000
2004
2008
2012
2016
Remaining life expectancy at age 75
Women
Men
Years
0
2
4
6
8
10
12
14
2000
2004
2008
2012
2016
Total population
Myocardial infarction
Ischemic stroke
Hemorrhagic stroke
Hip fracture
Colon cancer
Lung cancer
Breast cancer
