# Supplementary material for: Trends in life expectancy: did the gap between the healthy and the ill widen or close?
Source: BMC Med. 2020 Mar 20;18:41. doi: 10.1186/s12916-020-01514-z (PMC7082956; doi:10.1186/s12916-020-01514-z)
Supplement: Supplementary file 4 — Additional file 4: Table S1. Number of men and women in the total population and in subpopulations with disease history as well as mean age of disease onset in 1998, 2008, and 2017. [file 12916_2020_1514_MOESM4_ESM.docx]

*Additional file 4: Table S1. Number of men and women in the total population and in subpopulations with disease history as well as mean age of disease onset in 1998, 2008, and 2017*

|  | **Women** | | |
| --- | --- | --- | --- |
|  | **1998** | **2008** | **2017** |
| Total population | 927,767 | 956,697 | 1,105,539 |
| Myocardial infarction, n (%) | 45,223 (4.9%) | 52,604 (5.5%) | 47,097 (4.3%) |
| Age at disease onset, mean (SD) | 75.2 (8.2) | 76.0 (8.7) | 74.7 (8.8) |
| Ischemic Stroke, n (%) | 43,038 (4.6%) | 53,628 (5.6%) | 52,668 (4.8%) |
| Age at disease onset, mean (SD) | 77.4 (7.5) | 77.2 (8.2) | 76.2 (8.7) |
| Hemorrhagic Stroke, n (%) | 6,155 (0.7%) | 6,522 (0.7%) | 6,561 (0.5%) |
| Age at disease onset, mean (SD) | 76.0 (7.9) | 76.2 (8.6) | 75.7 (9.2) |
| Hip Fracture, n (%) | 64,957 (7.0%) | 66,840 (7.0%) | 61,588 (5.6%) |
| Age at disease onset, mean (SD) | 79.9 (7.8) | 79.7 (8.4) | 79.5 (9.1) |
| Colon Cancer, n (%) | 10,397 (1.1%) | 14,303 (1.5%) | 18,446 (1.7%)^a^ |
| Age at disease onset, mean (SD) | 74.2 (7.5) | 74.2 (7.8) | 74.0 (7.9)^a^ |
| Lung Cancer, n (%) | 1,996 (0.2%) | 3,493 (0.4%) | 5,975 (0.5%)^a^ |
| Age at disease onset, mean (SD) | 71.9 (6.5) | 72.2 (6.9) | 72.1 (6.7)^a^ |
| Breast Cancer, n (%) | 31,430 (3.4%) | 42,036 (4.4%) | 56,337 (5.1%)^a^ |
| Age at disease onset, mean (SD) | 71.2 (7.5) | 70.4 (7.7) | 69.8 (7.4)^a^ |
|  | **Men** | | |
|  | **1998** | **2008** | **2017** |
| Total population | 687,096 | 760,695 | 945,753 |
| Myocardial infarction, n (%) | 67,372 (9.8%) | 74,386 (9.8%) | 78,532 (8.3%) |
| Age at disease onset, mean (SD) | 71.7 (7.4) | 71.9 (8.0) | 71.0 (7.7) |
| Ischemic Stroke, n (%) | 41,441 (6.0%) | 51,816 (6.8%) | 57,598 (6.1%) |
| Age at disease onset, mean (SD) | 74.3 (7.4) | 73.9 (7.9) | 73.0 (8.0) |
| Hemorrhagic Stroke, n (%) | 6,313 (0.9%) | 7,231 (1.0%) | 7,800 (0.8%) |
| Age at disease onset, mean (SD) | 73.0 (7.2) | 72.7 (7.9) | 72.3 (8.1) |
| Hip Fracture, n (%) | 19,629 (2.9%) | 21,857 (2.9%) | 23,597 (2.5%) |
| Age at disease onset, mean (SD) | 77.8 (8.1) | 77.9 (8.7) | 77.4 (9.2) |
| Colon Cancer, n (%) | 8,346 (1.2%) | 12,023 (1.6%) | 16,537 (1.8%)^a^ |
| Age at disease onset, mean (SD) | 73.3 (7.8) | 73.3 (7.4) | 72.8 (7.4)^a^ |
| Lung Cancer, n (%) | 3,190 (0.5%) | 3,758 (0.5%) | 4,870 (0.5%)^a^ |
| Age at disease onset, mean (SD) | 72.5 (6.3) | 72.9 (6.7) | 72.8 (6.6)^a^ |
| *^a^ Data for year 2016. Proportions calculated using total population size 2016 (*1,096,240 women, 935,235 men)  *SD: Standard deviation* | | | |
